# Ophiostomatoid fungi synergize attraction of the Eurasian spruce bark beetle, *Ips typographus* to its aggregation pheromone in field traps

**DOI:** 10.3389/fmicb.2022.980251

**Published:** 2022-09-20

**Authors:** Anna Jirošová, Roman Modlinger, Jaromír Hradecký, Rajarajan Ramakrishnan, Kateřina Beránková, Dineshkumar Kandasamy

**Affiliations:** ^1^Faculty of Forestry and Wood Sciences, Czech University of Life Sciences, Prague, Czechia; ^2^Department of Biology, Lund University, Lund, Sweden

**Keywords:** spruce bark beetle, fungal VOCs, fusel alcohols and acetates, attraction, synergism, aggregation pheromones

## Abstract

Eurasian spruce bark beetle, *Ips typographus* is a destructive pest of the Norway spruce (*Picea abies*). Recent outbreaks in Europe have been attributed to global warming and other anthropogenic impacts. Bark beetles are guided by multiple complex olfactory cues throughout their life cycle. Male-produced aggregation pheromones, comprising 2-methyl-3-buten-2-ol and *cis*-verbenol, have been identified as the most powerful attractants for dispersing conspecifics. In addition to host trees, bark beetles interact with multiple organisms, including symbiotic ophiostomatoid fungi, which may promote beetle colonization success and offspring development. Previously, in a short-distance laboratory assay, we demonstrated that *I. typographus* adults are attracted to the volatile organic compounds (VOCs) produced by three symbiotic fungi: *Grosmannia penicillata*, *Endoconidiophora polonica*, and *Leptographium europhioides*. Furthermore, the abundant fusel alcohols and their acetates were found to be the most attractive odorants in the fungal VOC profile. In this study, using a long-distance field-trapping experiment, we analyzed the role of fungal VOCs as attractants for dispersing *I. typographus*. Two types of fungal lures were tested in combination with pheromones in traps: (1) live cultures of fungi grown on potato dextrose agar (PDA) and (2) dispensers containing synthetic fusel alcohols and their acetates in equal proportions. Subsequently, the composition of VOCs emitted from live fungal lures were analyzed. We found that the symbiotic fungi synergistically increased the attraction of beetles to pheromones in field traps and the attractiveness of live fungal lures depended on the fungal load. While one Petri dish with *E. polonica*, when combined with pheromones synergistically increased trapping efficiency, three Petri dishes with *L. europhioides* were required to achieve the same. The synthetic mix of fungal fusel alcohols and acetates improved the catch efficiency of pheromones only at a low tested dose. VOC analysis of fungal cultures revealed that all the three fungi produced fusel alcohols and acetates but in variable composition and amounts. Collectively, the results of this study show that, in addition to pheromones, bark beetles might also use volatile cues from their symbiotic fungi to improve tree colonization and reproductive success in their breeding and feeding sites.

## Introduction

Associations between insects and their ectosymbiotic fungi are common in terrestrial ecosystems. These fungi rely exclusively on insects for their dispersal and, in turn, provide a wide range of benefits to their vectors (Buser et al., 2014; Madden et al., 2018; Biedermann and Vega, 2020). The benefits provided by fungi include nutrition supplementation, detoxification of xenobiotics, defense of insect hosts from pathogens, and protection of insects from various abiotic conditions (Douglas, 2009; Oliver et al., 2014; Flórez et al., 2015; van den Bosch and Welte, 2017; Lemoine et al., 2020; Lehenberger et al., 2021). Although various cues are important for the behavioral ecology of insects, most insects rely on their sense of smell, that is, olfaction, to make crucial decisions in their life cycle. In some insect-fungus symbiotic associations, such as in the *Drosophila*-yeast system, where nutritional symbionts are acquired from the environment in each generation, volatile organic compounds (VOCs) produced by the fungi often serve as predominant cues to interact with the insects (Christiaens et al., 2014; Becher et al., 2018). Microbial VOCs act as semiochemicals for insects to elicit either an attraction or aversion behavior (Davis et al., 2013; Kandasamy et al., 2016).

The Eurasian spruce bark beetle, *Ips typographus* L. (Coleoptera: Curculionidae: Scolytinae), is an aggressive conifer pest that kills Norway spruce [*Picea abies* (L.) Karst. (Pinales: Pinaceae)] in the Eurasian forests. Recent warm and dry summers have promoted large-scale outbreaks of bark beetles, which have resulted in serious disruption of the spruce-dominated forest ecosystem in Europe (Huang et al., 2020; Hlásny et al., 2021; Netherer et al., 2021). Pioneer males select a suitable tree for colonization, construct nuptial chambers under the bark and infect the chamber walls with the microbes they carry. They attract potential mates and other males by releasing aggregation pheromones comprising *cis*-verbenol and 2-methyl-3-buten-2-ol, either to overwhelm the host tree defenses by a mass attack or for group foraging of the scarce resources. One to four females join with a male in the nuptial chamber, and each female constructs its oviposition tunnel in the inner bark, lays eggs, and inoculates symbiotic fungi in the bark (Biedermann et al., 2019). The occurrence of fungi, especially polyphyletic ophiostomatoid fungi, such as *Grosmannia penicillata* (Grosmann) Goid., *Endoconidiophora polonica* (Siemaszko) Z.W. de Beer, T.A. Duong and M.J. Wingf., *Leptographium europhioides* (E. F. Wright and Cain) M. Procter and Z.W. de Beer, and *Ophiostoma bicolor* R.W. Davidson and D.E. Wells, is common and abundant (Kirisits, 2007; Linnakoski et al., 2012). Ophiostomatoid fungi may assist beetles in colonizing healthy trees by exhausting their defense mechanisms, detoxifying host defense chemicals, and supplementing the beetles with essential nutrients that are crucial for their larval development and maturation (Bleiker and Six, 2007; François et al., 2009; Krokene, 2015; Wadke et al., 2016; Kandasamy et al., 2021).

A recent study employing a short-distance laboratory assay (Kandasamy et al., 2019) has shown that endogenous VOCs, especially fusel alcohols and acetates, produced by the symbiotic fungi, are behaviorally active and attract young adults to them. These findings suggested that immature or callow adults might utilize fungal VOCs to seek out nutritious fungi under the bark to feed on them for attaining sexual maturity. In particular, callow adults of *I. typographus* preferentially fed on *E. polonica*-, *G. penicillata*-, and *L. europhioides*-colonized spruce bark, over the uncolonized control diet. In short-range (ca. 13 cm) olfactometer assays, beetles were strongly attracted to the volatile profile of *E. polonica*, *G. penicillata*, and *L. europhioides* grown on potato dextrose agar (PDA), over the uncolonized agar. *Endoconidiophora polonica* volatile profile consisted mostly of fusel acetates and less fusel alcohols, whereas the volatile profiles of *G. penicillata* and *L. europhioides* contained mostly fusel alcohols and less fusel acetates and sesquiterpenes. Furthermore, in short-distance assays, the synthetic mix of the *E. polonica*-mimic blend and the equal-ratio mix of all fusel alcohols and acetates were attractive at low doses. *Ips typographus* detects fusel alcohols and their acetates through numerous broadly tuned olfactory neurons in their antennae. In addition to fusel alcohols and acetates, *G. penicillata* and *L. europhioides* have been reported to produce the *I. typographus* aggregation pheromone, 2-methyl-3-buten-2-ol *de novo*, spiroketals such as *exo*- and *endo*-brevicomin, and *trans*-conopthorin, when inoculated into the spruce bark (Zhao et al., 2015, 2019). *Exo*- and *endo*-brevicomins are pheromone components of several bark beetles, especially of the genus *Dendroctonus* (Keeling et al., 2021). However, the ecological relevance of fungi (i.e., attraction or repulsion of beetles), which produce semiochemicals for the bark beetles, remains unknown. In addition, it remains unknown whether the dispersing adult *I. typographus*, once it leaves its brood galleries to seek suitable host trees, would also respond to a blend of fungal VOCs, apart from its aggregation pheromone.

Previous studies have provided ample evidence of the attraction of diverse insect species to the live cultures of fungi in agricultural landscapes (Nout and Bartelt, 1998; El-Sayed et al., 2005; Davis et al., 2012; Davis and Landolt, 2013; Andreadis et al., 2015). These studies showed that the attraction of insects to fungal VOCs appears to be a common ecological phenomenon, probably because many insects depend on symbionts for their nutrition and development. Bark beetles have an intricate relationship with fungal symbionts, and the importance of fungal VOCs in bark beetle biology has only recently been recognized (Kandasamy et al., 2016). The relevance of the attraction of bark beetles to fungi or their complex bouquets under field conditions remains unknown for any bark beetle species. Only individual compounds found in the volatile profile of ophiostomatoid fungi have been tested in field trials, together with pheromones of different bark beetle species. For example, 2-phenylethanol reduced the attraction of *Dendroctonus frontalis* and *Dendroctonus ponderosae* to their corresponding aggregation pheromones (Pureswaran et al., 2000; Sullivan et al., 2007). However, *I. typographus* did not exhibit any behavioral response when 2-phenylethanol was tested in combination with its aggregation pheromone (Schlyter et al., 1987).

In this study, VOCs from ophiostomatoid fungi were investigated as synergistic attractants for *I. typographus* pheromones. The main objective of this study was to test whether fungal VOCs could improve the attractiveness of *I. typographus* pheromones in field traps. Two types of fungal VOC baits were used together with pheromones in traps: live cultures of fungi on PDA and a synthetic blend of fusel alcohols and their acetates having different release rates. Our findings clearly showed that symbiotic fungal VOCs synergize the attraction of dispersing adult *I. typographus* to its aggregation pheromones.

## Materials and methods

### Preparation of fungal culture lures

Three common symbionts of *I. typographus*-*E. polonica*, Ep (MPICE-2014-1 isolated in 2014 from Gotha, Germany); *G. penicillata*, Gp [NFRI-2006-209/44/2 isolated in 2006 from Kronoberg (Växjö), Sweden]; and *L. europhioides*, Le [NFRI-1990-119/20 isolated in 1990 from Nord-Trøndelag (Namsskogan), Norway], were used in this study (Kandasamy et al., 2019). Under sterile conditions, fungi were cultured on PDA (VWR, Leuven, Belgium) in plastic Petri dishes (9 cm diameter, 2 cm wall thickness) and incubated at 25°C for 4 days in the laboratory, before being transferred to the field. In 4-day-old fungal culture Petri dishes, 30 uniformly placed holes (each 2 mm in diameter) were made on the lids (1 hole/2.12 cm^2^ of lid) using a soldering unit. Later, the Petri dishes were temporarily sealed using a Parafilm and carried to the field in a cold box.

### Preparation of synthetic volatile organic compounds dispensers

A mixture of fungal VOCs containing fusel alcohols and acetates: 3-methyl-1-butanol (Sigma- Aldrich, 95%), 2-methyl-1-butanol (Sigma-Aldrich, 9%), 3-methyl-1-butyl acetate (Sigma-Aldrich, 95%), 2-phenylethanol Sigma-Aldrich, 99%), 2-methyl-1-butyl acetate (Sigma-Aldrich, 99%), and 2-phenylethyl acetate (Sigma-Aldrich, 98%) was prepared at a ratio of 1:1:1:1:1:1 (v/v). The actual release rates of the dispensers were determined in the laboratory under ambient conditions (25°C, 0.5 m/s airflow) using the gravimetric method (Table 1). Three replicates of each dispenser type were weighed for 10 days at regular intervals, and weight loss was recorded to the nearest 0.0001 g using a fine balance. The average release rate for each dispenser type was calculated based on the average weight loss over 24 h. Additionally, the average release rate of dispensers, under natural field conditions, was determined using the gravimetric method by periodically weighing the dispensers used in the field.

**TABLE 1:** Design and release rates of artificial dispensers containing the mixture of fusel alcohols and acetates.

Compounds	Release rate (mg/day) ± SD		No. of dispensers	Dispenser design
	
	Laboratory	Field		
Synthetic fungal VOCs mix^†^				
Mix (low)	0.34 ± 0.04	0.3 ± 0.1	1	Glass vial with a syringe hole on septa^†⁣†^.
Mix (Medium)	1.7 ± 0.2	1.1 ± 0.3	1	PE/Aluminum sachet with 0.5 mm hole^†⁣†⁣†^
Mix (High)	4.2 ± 0.78	2.9 ± 1.1	2	PE/Aluminum sachet with 2 mm hole ^†⁣†⁣†^
*Ips typographus* pheromone				
2-methyl-3-buten-2-ol	42.2 ± 20	62 ± 25	1	731 vial; 2 mm hole on cap^†⁣†⁣†⁣†^
*cis*-verbenol	1.5 ± 0.8	1.2 ± 1.0	1	731 vial; 9 mm hole on cap^†⁣†⁣†⁣†^

^†^3-methyl-1-butanol, 2-methyl-1-butanol, 3-methyl-1-butylacetate, 2-phenylethanol, 2-methyl-1-butyl acetate, 2-phenylethyl acetate in ratio 1:1:1:1:1:1 (v/v). ^†⁣†^2 ml glass vial with a PTFE silicone septum, syringe 22 gauge (Agilent, CA, United States). ^†⁣†⁣†^Cellulose sponge (6 cm × 4 cm × 0.25 cm) filled with 2 ml of volatile organic compounds (VOCs) mix, sealed in 0.1 mm thickness PE film. The adsorbent with VOCs was sealed in an aluminum sachet (7 cm × 5 cm) with the hole at the center of one side. ^†⁣†⁣†⁣†^Closed PE vial (731, Kartell, Catania, Italy) with a hole on the cap.

### Study site and overall field experimental setup

**FIGURE 1:**
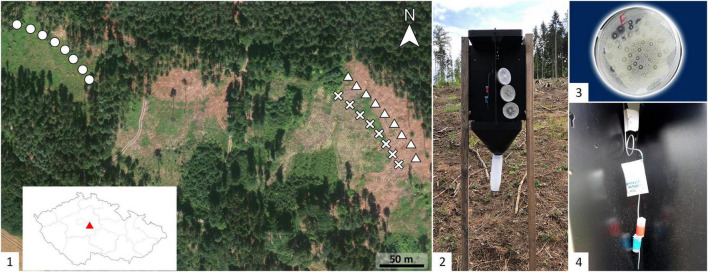
Experimental field site and trap design. (1) Location within the Czech Republic and arrangement of traps for live fungal bait experiment in 2019 (circles) and in 2020 (crosses); synthetic volatile organic compounds (VOCs) bait experiment in 2020 (triangles). (2) Cross-vane trap baited with Petri dish dispensers containing actively growing fungus and *I. typographus* pheromone components, 2-methyl-3-buten-2-ol and *cis*-verbenol. Close-up view of (3) perforated Petri dish dispenser, which was hung in the trap together with pheromone components, and (4) dispenser with synthetic fungal compounds and pheromones.

The study was conducted at the Czech University of Life Sciences, Prague-owned School Forest Enterprise in Kostelec and Černými Lesy, in the Czech Republic for two consecutive years, 2019 and 2020 in two blocks. In 2019, the first block of the experiment using fungal cultures was run from 25 April to 25 May in a 100 m long, 60 m wide clear cut created in 2018 in the Norway spruce forest (49.9339142N, 14.8726833E). Nine cross-vane traps (Ecotrap, Fytofarm, Bratislava, Slovak Republic) were placed in a single row, 30 m southwest of the open forest edge [Figure 1-(1)]. Traps were fixed on wooden poles that were placed 1.5 m above the ground, and the distance between the traps was 15 m.

In 2020, the second block of the fungal culture experiment and analysis of baits prepared from synthetic fungal VOCs were held from 18th May to 1st June in 150 m long and 100 m wide clear cuts made during the winters of 2019/2020 (location:49.9333822N, 14.8773022E). Nine traps were placed in a single row, 30 m from the forest edge. Eight traps with synthetic baits were placed in a second row running parallel to the forest edge, 50-m distance from the forest edge, and 20 m from the traps for the fungal culture experiment [Figure 1-(1)].

**FIGURE 2:**
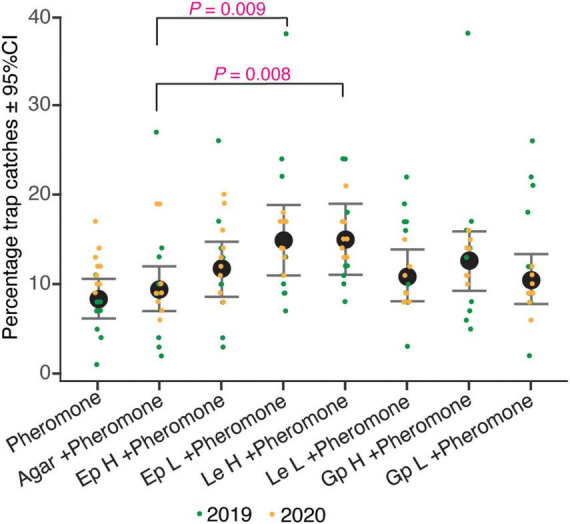
Percentage trap catches of *I. typographus* in pheromone traps baited with various loads of different fungal cultures. pheromone: *I. typographus* pheromone consists of 2-methyl-3-buten-2-ol and *cis*-verbenol with a release rate of 42 and 1.5 mg/day, respectively; agar, perforated Petri dish (90 mm in diameter) containing only potato dextrose agar (PDA); Ep, *Endoconidiophora polonica*; Le, *Leptographium europhioides*; Gp, *Grosmannia penicillata* grown on PDA for 4–7 days; H, high load of fungus culture (three Petri dishes); L, low load of fungus culture (one Petri dish). Whiskers represent 95% confidence intervals (CI), black dots are the predicted mean of combined year percentage trap catches generated by the linear mixed model and colored dots are original values from different years.

**FIGURE 3:**
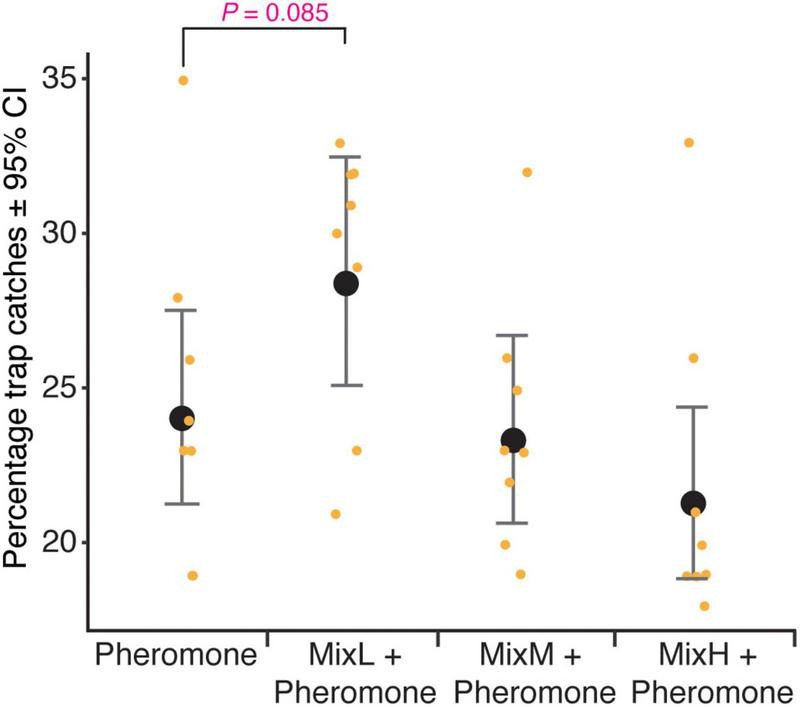
Percentage trap catches of *Ips typographus* in pheromone traps baited with dispensers containing a mixture of 3-methyl-1-butanol, 2-methyl-1-butanol, 3-methyl-1-butylacetate, 2-phenylethanol, 2-methyl-1-butylacetate, and 2-phenylethylacetate in equal volume ratio 1:1:1:1:1:1 (v/v). Dispensers denoted as MixL, MixM, and MixH have release rate of 0.34, 1.7, and 8.4 mg/day, respectively; pheromone: pheromone consisting of 2-methyl-3-buten-2-ol and *cis*-verbenol with a release rate of 42 and 1.5 mg/day, respectively. Whiskers represent 95% CI, black dots are the predicted mean generated by the linear regression model and orange dots are original values.

**FIGURE 4:**
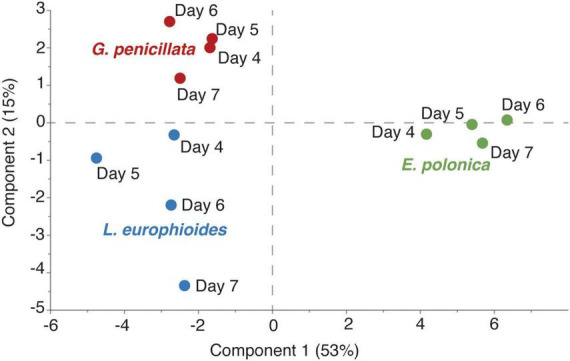
Partial least square-discriminant analysis (PLS-DA) score plot of headspace volatile organic compounds (VOCs) collected at different time points after inoculation with three fungi grown on potato dextrose agar (PDA). *X*- and *Y*-axes represent scores of components 1 and 2 respectively, obtained from PLS-DA analysis. *Endoconidiophora polonica* (green); *Leptographium europhioides* (blue); *Grosmannia penicillata* (red). SIMCA 17 (Sartorius Stedim, Sweden) software was employed using normalized (constant sum) data.

Petri dishes containing the fungal cultures and perforated lids, prepared as described above, were baited in the barrier traps by hanging them vertically on a metal wire with the perforated side exposed to the air. Synthetic baits, containing the pheromone components (62 mg/day of 2-methyl-3-buten-2-ol, 1.2 mg/day of *cis*-verbenol), were suspended on a metal wire, parallel to the fungal culture [Table 1, Figure 1-(2,3)]. Different loads of the fungal cultures were tested in combination with pheromones. Pheromone alone and pheromone with fungus-free PDA were used as the controls. The “high” load fungal culture contained three perforated Petri dishes, and the “low” load culture contained only one perforated Petri dish. Dispensers with a mixture of synthetic VOCs with release rates of 8.4, 1.7, and 0.34 mg/day were tested, both in combination with pheromones and individually in the same experiment [Figure 1-(4)].

In both 2019 and 2020, the same block design was used to test the efficiency of fungal lures in improving the attractiveness of pheromones. Each block contained nine baits (empty, agar + pheromone, Ep H + pheromone, Ep L + pheromone, Le H + pheromone, Le L + pheromone, Gp H + pheromone, and Gp L + pheromone), which were relocated (replication) nine times in a Latin square design. Old fungal culture lures were replaced every 4 days with fresh 4-day-old fungal cultures, as the fungal cultures reached a stationary growth phase after 7 days. In 2020, additionally, the synthetic VOCs lures experiment was conducted in a block design with eight baits (empty, pheromone, Mix L + pheromone, Mix M + pheromone, Mix H + pheromone, MixL, MixM, and MixH), which were rotated eight times. The daily mean temperature during the experimental period was 11°C in 2019 and 13°C in 2020 (Supplementary Figure 1), and the mean temperature at noon was between 19 and 25°C. During each rotation, the collection jars were emptied and adult *I. typographus* were stored in Falcon tubes containing >99% ethanol (v/v). The specimens were counted later in the laboratory and sex was determined by dissection. In catches with more than 50 beetles, 50 were randomly selected for sex determination. The relative catch efficiency of the traps in percentage was calculated as the number of beetles caught in a single treatment to the sum of all bark beetles caught during a single rotation.

### Chemical composition of headspace volatile organic compounds from fungal culture lures

Petri dishes containing the fungal cultures were prepared as described above. Thirty holes (2 mm in diameter) in the lid were made on the day of the first analysis of the VOCs. Petri dishes were kept in an incubator at 25°C, except at the time of headspace VOC collection. Dynamic headspace VOCs from fungal cultures were collected using a pull system in 2 L glass jars, closed by a silicone stopper with two pre-made holes. At 4-, 5-, 6-, and 7-days post-inoculation, two Petri dishes of each fungus were placed in a glass jar, a cartridge containing Tenax TA sorbent (SKC, PA, USA) was connected to the outlet hole, and the air passing through inlet hole was filtered using a similar cartridge. The sucking pocket pump with an airflow of 100 ml/min was attached to the sorbent cartridge in the outlet tube, and the headspace VOCs were collected for 2 h at 25°C. The cartridges containing the VOCs were extracted five times with 200 μl hexane. The same two Petri dishes per fungus were used repeatedly for headspace VOC collection at 5-, 6- and 7-days post-inoculation.

VOCs were analyzed using two-dimensional gas chromatography coupled with time-of-flight mass spectrometry (GC × GC-TOF-MS) (Leco Pegasus 4D, Leco, MI, USA) in a single-dimension mode. Injection volume was set at 1 μl and a programmed temperature vaporizing injector (PTV) was used in cold split-less (20°C) injection mode and then heated to 280°C at 8°C/s. For compound separation, a 30 m (0.25 mm i.e., 0.25 μm film thickness) Rxi-5MS (Restec, PA, USA) column was used. The oven temperature program was set at 40°C for 2 min, then ramped up at 5°C/min to 120°C, then at 10°C/min to 200°C, and finally at 20°C/min to 280°C and held for 2 min. The mass spectrometer was operated in the mass range of 35–500 m/z with an acquisition speed of 10 Hz. The total GC run time was about 32 min. The VOC concentration was calculated using external calibration curves generated from commercially available pure 3-methyl-1-butanol, 2-methyl-1-butanol, 3-methyl-1-butyl acetate, 2-phenylethanol, 2-methyl-1-butyl acetate, and 2-phenylethyl acetate. For confidence interval, relative standard deviation (2RSD) (Ep, day 5, *n* = 6) was used. Other compounds discussed in the non-target VOCs analysis were identified based on the spectral similarity with the NIST mass spectral library (NIST 2017), and this identification was supported by a comparison of measured and published retention indices.

### Statistical analyses

R software version 4.1.3 (R Core Team, 2022) was used for statistical analyses. Linear mixed-effect models were used to analyze data from the fungal culture lure experiment with the year added as a random variable. The dependent variables were the relative number of trapped beetles, and the independent variables were different fungal culture lures in various quantities. The dependent variable was logarithmically transformed, and the outcome of the best model followed Gaussian distribution. The comparison of means was done using the “nlme” function for R (Pinheiro et al., 2022). The model selection and validation were performed using the protocol from Zuur and Ieno (2016), using Akaike information criterion (AIC) and Q-Q-plot residuals. The null hypothesis of factor non-significance was rejected at the standard level of α = 0.05. Treatment contrast was applied to compare the significance of all factors against agar + pheromone catches. A linear model was used to analyze data from the synthetic VOCs experiment. The dependent variable was the relative number of trapped beetles, the proportion of males, and the independent variables were different doses of VOCs mix. The dependent variable was logarithmically transformed, and the outcome of the best model followed Gaussian distribution. Treatment contrast was applied to compare all factors against pheromone-only catches. Owing to the lack of significance in the case of the proportion of males, only *F*-test was tested.

## Results

### Ophiostomatoid fungi increase the attractiveness of pheromone baits in field traps

The total number of *I. typographus* adults captured in 2019 was 14,071, and that in 2020, was 4,889. (Supplementary Tables 1, 2). Trap catches varied much throughout each study season, with 1–3,074 beetles per trapping day in 2019 and 24–131 beetles in 2020. The variability in absolute catches was caused by fluctuations in weather and beetle activity (Supplementary Figure 1).

The relative number of beetles caught in traps containing different fungal culture lures were significantly different (ANOVA, *F* = 2.87, df = 7, *P* < 0.01; Supplementary Table 3). The four fungal culture treatments, namely the high and low doses of *E. polonica* (Ep H and Ep L), high doses of *L. europhioides* (Le H), and *G. penicillata* (Gp H) had higher relative catches, compared to that in the control, in both the years (Figure 2). However, only the combined relative catches of Ep L and Le H in both the years were statistically significant (Ep L, *t* = 2.65, *P* < 0.01; Le H, *t* = 2.69, *P* < 0.01) (Table 2). For Gp H and Le H, the variant with the highest fungal dose always achieved higher catches, and for Ep L, the variant with the lower fungal dose had higher catches, compared to that in the control.

**TABLE 2:** Field attraction of *Ips typographus* to ophiostomatoid fungi. The table shows the results of contrast comparison estimated from a linear mixed model, log (Relative catches of *I. typographus*) ∼ Baits + (1| Year). Baits contain aggregation pheromone in combination with various loads of different live fungal cultures.

Baits	Estimate	Std. error	*t*-value	Pr(>| t|)
Agar + pheromone^†^ (Intercept)	2.212	0.138	16.024	**0.000**
Pheromone	−0.123	0.170	−0.725	0.469
Ep H + pheromone	0.206	0.170	1.207	0.229
Ep L + pheromone	0.452	0.170	2.650	**0.009**
Le H + pheromone	0.459	0.170	2.689	**0.008**
Le L + pheromone	0.145	0.170	0.854	0.394
Gp H + pheromone	0.281	0.170	1.650	0.101
Gp L + pheromone	0.108	0.170	0.635	0.526

Ep, Endoconidiophora polonica; Le, Leptographium europhioides; Gp, Grosmannia penicillata; H, high load; L, low load; Pr(>| t|), p-value for t statistic; Boldface numbers show significant effects at the level 0.05 when compared against control treatment (Agar + pheromone)^†^.

In the 2019 experiment, the proportion of females was at an average of 78 ± 10%, and in 2020, it was at 81 ± 6%, from all the catches. The proportion of female catches in the different fungal cultures was not significantly different from that of the controls and among fungal species (ANOVA, *F* = 0.225, df = 3, *P* = 0.88).

### Synthetic fungal volatile organic compounds containing fusel alcohols and their acetates improve the attractiveness of pheromones

A total of 3,514 beetles were caught in this experiment. The absolute number of beetles caught in the traps varied from 42 to 211 (Supplementary Tables 4, 7). There was a statistically significant difference between the relative number of bark beetles caught by the different types of dispensers tested in combination with pheromones (ANOVA, *F* = 3.298, df = 3, *P* < 0.05) (Supplementary Table 5). The relative catches decreased with increasing release rates of the fungal VOC mixture. The highest relative catch was recorded in the dispenser with the lowest release rate (MixL); however, the difference in catches between MixL and pheromone-only bait was not statistically significant (contrast *t*-test, *P* = 0.09, Supplementary Table 6) (Figure 3). The proportion of females, caught in all the dispensers in combination with pheromones, was 75 ± 6% and was not significantly different among them (ANOVA, *F* = 0.225, df = 3, *P* = 0.88).

The absolute number of beetles caught in synthetic VOC dispensers without pheromones ranged from 0 to 6. The empty control traps did not catch any beetle. Only the MixH bait caught some beetles, but the catches were meager, with a maximum of six (Supplementary Tables 8, 9).

### Fusel alcohols and their acetates dominate the VOC profile of fungal culture lures

Time-series headspace VOCs were collected from different fungi grown on PDA and analyzed using GC-TOF-MS. The partial least square-discriminant analysis (PLS-DA) score plot showed that nearly 68% variation in the volatile profiles was explained by the first two components and the model quality parameters (R2Xcum = 0.80, R2Ycum = 0.94, and Q2cum = 0.75) showed that there was a clear distinction in the VOC profiles of *E. polonica*, *G. penicillata*, and *L. europhioides* (Figure 4). For *E. polonica*, the most distinct feature was the abundance of acetate acetates (especially isobutyl acetate). In *G. penicillata* and *L. europhioides*, the most distinct factors were the abundance of methyl isovalerate, unknown sesquiterpenes, propyl acetate, and 3-methyl-1-butyl acetate. As fusel alcohols and their acetates are biologically active, we quantified these VOCs in the headspace of the Petri dish dispensers. In *E. polonica*, the release rates of 3-methyl-1-butyl acetate and 2-methyl-1-butyl acetate ranged from 1,000 to 5,500 ng/h/Petri dish, and from 200 to 1,500 ng/h/Petri dish, respectively, at an 8:1 ratio on day 4 and 3:1 on day 7. In *L. europhioides*, acetates were detectable only from day 6, with a 300 ng/h/Petri dish of 3-methyl-1-butyl acetate and 30 ng/h/Petri dish of 2-methyl-1-butyl acetate at a 9:1 ratio. In *G. penicillata*, only 3-methyl-1-butyl acetate was detected on day 6 in a 35 ng/h/Petri dish.

**FIGURE 5:**
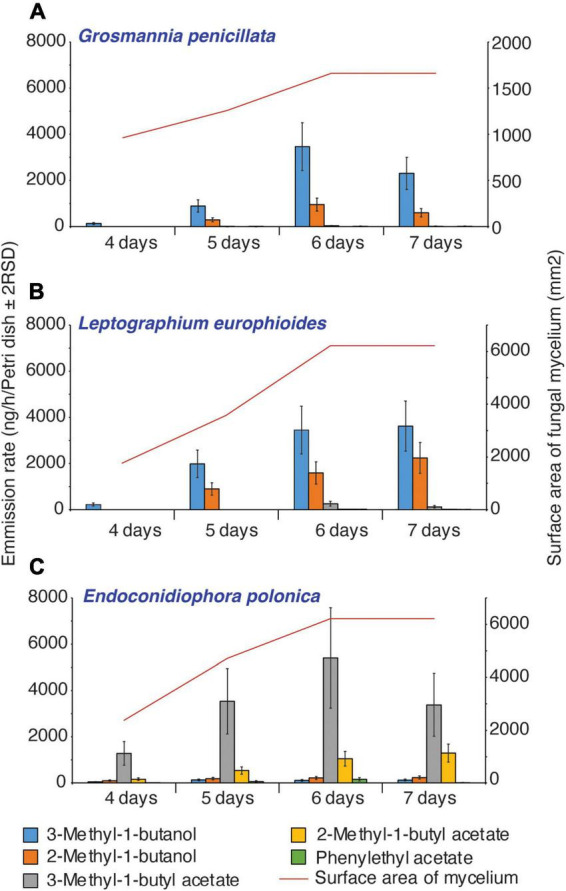
Emission rate of fusel alcohols and their acetates from perforated Petri dish dispensers inoculated with different fungi in relation to the surface area of fungal mycelium at the time of sampling. Error bars represent relative standard deviation (2RSD) (Ep, day 5, *n* = 6).

Fusel alcohols, such as 3-methyl-1-butanol and 2-methyl-1-butanol, were the most abundant in cultures of *L. europhioides* and *G. penicillata*. The release rate of 3-methyl-1-butanol in *L. europhioides* varied from 200 ng/h/Petri dish on day 4 to 3,500 ng/h/Petri dish on day 7, and in *G. penicillata* ranged from 100 ng/h/Petri dish on day 4 to 3,500 ng/h/Petri dish on day 6 (Figure 5). The release of 2-methyl-1-butanol was lower for both the fungi. The ratio of these compounds was approximately 2:1 for *L. europhioides* and 4:1 for *G. penicillata*, in the recorded period. In *E. polonica*, the release rate of fusel alcohols was low at 50–130 ng/h/Petri dish of 3-methyl-1-butanol and 100–250 ng/h/Petri dish of 2-methyl-1-butanol at a 0.5:1 ratio (Figure 5). Additionally, *L. europhioides* produced methyl isovalerate. All fungi released low amounts of 2-phenylethyl acetate. The release rate of fusel alcohols and their acetates reached a maximum on day 6, and then either decreased in *E. polonica* and *G. penicillata* or remained constant in *L. europhioides* until day 7. The release rate of VOCs was proportional to the growth rate of fungi. Fungi actively grew until they reached the edge of the dish and attained their maximum surface area (mm^2^) on day 6 (Figure 5).

Other VOCs, such as sesquiterpene hydrocarbons, were produced by all fungi in relatively lower amounts. For example, β-caryophyllene levels in *L. europhioides* reached a maximum on day 7. In general, *L. europhioides* released several sesquiterpenes in higher amounts, compared to the other two fungi (Supplementary Figure 2).

## Discussion

We found that VOCs emitted from live fungal cultures synergize the attraction of *I. typographus* to its aggregation pheromones in barrier traps. To our knowledge, this is the first study to show that VOCs from fungal cultures synergize the attraction of bark beetles with their pheromones under natural field conditions. The three common fungal associates of *I. typographus*-*E. polonica*, *G. penicillata*, and *L. europhioides* emit distinct volatile cues dominated by fusel alcohols and their acetates when grown on PDA (Figures 4, 5) (Kandasamy et al., 2019). Traps baited with low VOC-emitting *E. polonica* culture and high VOC-emitting *L. europhioides* culture, together with pheromones, significantly increased the attraction of beetles to the pheromones, compared to the traps with pheromones alone. Additionally, the synthetic mix of fusel alcohols and acetates produced by fungi at a low release rate increased the attraction of beetles to pheromones. Collectively, these results show that fungal VOCs could play a previously overlooked yet crucial role in host colonization of beetles by synergizing pheromone attraction in conspecifics.

The attractive load of fungal cultures varies among the species. *Endoconidiophora polonica* released high amounts of fusel acetate and low amounts of fusel alcohol, whereas *L. europhioides* and *G. penicillata* released high amounts of fusel alcohol and low amounts of acetate ester. Our field-trapping experiment clearly showed that low loads of *E. polonica* and high loads of *L. europhioides* cultures were attractive. We also showed that there is moderate evidence that a synthetic blend of fungus-produced fusel alcohols and acetates increased the attraction of pheromones only at low doses. These results agree with those of a previous study (Kandasamy et al., 2019), in which an *E. polonica* mimic blend and a synthetic blend of alcohols and acetates were attractive at lower doses in a short-distance laboratory trap bioassay. Based on these results, it appears that fusel acetates with low release rates are the main active components in the attractive blend of fungal VOCs. As *E. polonica* releases a large amount of acetate acetates, attraction occurs at a lower load in field traps. However, as *L. europhioides* releases only a low amount of acetate acetates, a higher load is necessary. Unexpectedly, the *G. penicillata* culture did not significantly increase the attraction of pheromones, even though it is an important fungal symbiont of *I. typographus* (Zhao et al., 2018; Tanin et al., 2021). In this study, the analysis of the volatile profile of *G. penicillata* revealed only trace amounts of acetate acetates, a finding which is in contrast with a previous study in which the same isolate produced a substantial amount of acetate acetates (Kandasamy et al., 2016, 2019). It could be attributed to a well-documented fact that some fungal pathogens maintained in the laboratory degenerate over time or exhibit phenotypic plasticity due to a lack of selection pressure from the host substrate (Krokene and Solheim, 2001).

We found that a lower load of *E. polonica* culture is sufficient to increase the attraction of pheromones; therefore, we speculate that beetles can successfully colonize the host tree with a lower spore load of *E. polonica*. In fact, the abundance of *E. polonica* isolated from the beetles and their breeding galleries, and the proportion of beetles that carry this fungus have been reported to be low in most sampling locations across Europe (Linnakoski et al., 2012, 2016; Giordano et al., 2013). *E. polonica* is the primary invader of host trees and is better suited to grow in fresh tissues and to rapidly colonize the sapwood faster than other fungal symbionts of *I. typographus* (Solheim, 1991, 1992). *Endoconidiophora polonica* is one of the most virulent pathogens and has been shown to kill the host trees, following an artificial inoculation (Horntvedt et al., 1983; Wadke et al., 2016). When the beetle population is endemic, the colonization of the whole tree by *I. typographus* usually lasts several weeks because of the low beetle density in the vicinity (Toffin et al., 2018). In this scenario, *E. polonica* inoculated by a few early colonizers should be sufficient to be established in the bark because of its fast growth rate and rapid colonization of fresh tissues without any competition from other microbes (Solheim, 1992). In addition, fungal VOCs have been detected in infected spruce tissues from the very first day of infection and are shown to increase exponentially thereafter (Zhao et al., 2015; Kandasamy et al., 2021). Therefore, it is very likely that VOCs produced by *E. polonica* are released simultaneously with the aggregation pheromone of the beetle, which can synergistically increase the attraction of conspecifics to colonize the tree.

The synthetic blend of fungal VOCs alone did not capture many bark beetles. Remarkably few beetles were found in traps baited with a high dose of fungal VOCs mix (Supplementary Tables 8, 9). In addition to fusel acetates, the fungal VOCs mix also contained 3-methyl-1-butanol and 2-methyl-1-butanol, which are structurally similar to the beetle pheromone, 2-methyl-3-buten-2-ol. Notably, 2-methyl-1-butanol has been found to elicit a strong secondary response in the olfactory neurons of *I. typographus*, that primarily respond to 2-methyl-3-buten-2-ol (Kandasamy et al., 2019). Thus, catches with high doses of fungal VOCs mix alone could arise because of secondary activation of pheromone neurons by high amounts of fusel alcohols. Another explanation could be that the aggressive bark beetles, such as *I. typographus*, which are specialized in a single conifer species, respond to fungal VOCs only in the presence of specific background signals, such as pheromones or host tree VOCs. Indeed, we found increased trapping of *I. typographus* in the fungal baits only in the presence of pheromones. In contrast, fungal VOCs alone were sufficient to trap several generalist insects that forage on multiple plant species in agricultural landscapes (Davis and Landolt, 2013; Andreadis et al., 2015). Interestingly, many fungi associated with different bark beetle species share similar components in their volatile profiles and mainly consist of fusel alcohols and/or their acetates (Cale et al., 2016; Kandasamy et al., 2016). A recent study showed that at a short distance, adult *I. typographus* did not discriminate between its fungi and the fungi associated with the North American spruce bark beetle, *Dendroctonus rufipennis*, likely because of their similar volatile profiles (Tanin et al., 2021). Therefore, it is likely that *I. typographus* is evolutionarily adapted to respond to fungal VOCs from long distances only in combination with pheromones to avoid cross-attraction to non-symbiotic fungi vectored by a different bark beetle. As pheromones are usually species-specific, their combination with fungal VOCs represents a reliable signal for dispersion of the beetles, indicating the presence of conspecifics and a suitable fungal symbiont.

Higher catches in traps containing a combination of pheromones and fungal VOCs indicate that fungal VOCs play an important role in beetle aggregation and colonization. Fusel alcohols and their acetates are produced by the fungi by the metabolism of amino acids via the Ehrlich pathway, which is present only in microbes (Hazelwood et al., 2008). It has been suggested that fungi release fusel alcohols and their acetates to signal the insects about the nutritional quality of the diet comprising of sugars and amino acids and, in turn, insects act as dispersal agents to transport the fungal spores to new substrates/host (Saerens et al., 2010; Madden et al., 2018). It is noteworthy that most fungi that release fusel alcohols and acetates are often nutritional symbionts of their corresponding mutualistic insect partners, and insects are adapted to locate their symbiotic partners through numerous broadly tuned olfactory receptors that detect fungal VOCs (Davis et al., 2013; Christiaens et al., 2014; Mansourian and Stensmyr, 2015; Becher et al., 2018). Based on the current and a previous study, we clearly showed that bark beetles, such as *I. typographus*, are attracted to their fungal symbionts both in the field and laboratory conditions. This provides another example to the increasing body of evidence that insect-fungus relationships are mediated by fungus-produced VOCs. Furthermore, as the association between *I. typographus* and its fungal symbionts is still under debate, the results of this study indicate that there is a potential mutualistic relationship between *I. typographus* and its fungal symbionts.

The volatile profile of fungi depends on substrate quality and environmental conditions. In this study, fungi were grown on an artificial substrate, and only the VOCs endogenously produced by them were investigated. However, in nature, fungi colonize the bark phloem and sapwood with a distinct nutrient profile containing high amounts of defense chemicals such as terpenes and phenolics. Recent studies showed that *I. typographus*—symbiont fungi metabolize the terpenes and phenolics and release a suite of VOCs that function as semiochemicals for *I. typographus* (Kandasamy et al., 2021; Jirošová et al., 2022). Future studies should investigate the synergistic effect of the fungal VOCs on pheromone attraction by inoculating the fungal cultures on natural substrates, such as spruce bark phloem and sapwood. We acknowledge the limitations of testing pure single-species cultures under field conditions. In the breeding galleries constructed by the beetles, several closely related species of ophiostomatoid fungi and yeasts co-inhabit the phloem of the bark (Giordano et al., 2013; Linnakoski et al., 2021). Therefore, one approach would be to analyze mixed microbial cultures in their natural growth substrates together with pheromones in field traps. Such efforts could provide new combinations of semiochemicals that could be added to the current practices of the management of bark beetles.

## Data availability statement

The original contributions presented in this study are included in the article/Supplementary material, further inquiries can be directed to the corresponding author.

## Author contributions

AJ and DK conceived the project, designed the experiments, and wrote the manuscript with inputs from all the co-authors. AJ, KB, JH, and RR performed the experiments. RM analyzed the data. All authors approved the final version of the manuscript.
