# Supplementary figures and images for: Ophiostomatoid fungi synergize attraction of the Eurasian spruce bark beetle, Ips typographus to its aggregation pheromone in field traps
Source: Front Microbiol. 2022 Sep 20;13:980251. doi: 10.3389/fmicb.2022.980251 (PMC9530181; doi:10.3389/fmicb.2022.980251)

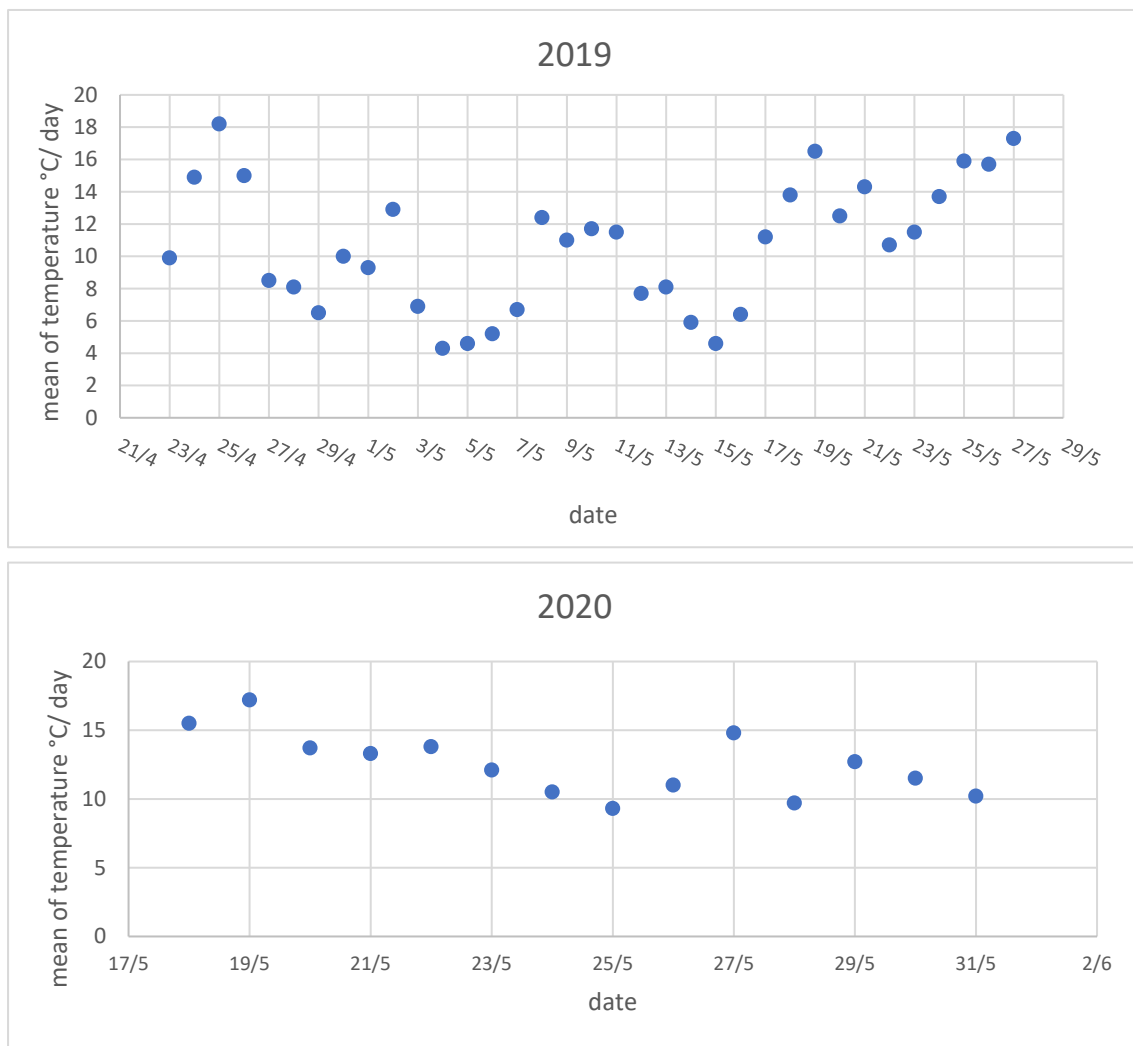

**Figure S1:** Daily mean air temperature during experimental periods

Supplement: Supplementary file 10 [file Image_1.pdf]
