# Supplementary material for: Ophiostomatoid fungi synergize attraction of the Eurasian spruce bark beetle, Ips typographus to its aggregation pheromone in field traps
Source: Front Microbiol. 2022 Sep 20;13:980251. doi: 10.3389/fmicb.2022.980251 (PMC9530181; doi:10.3389/fmicb.2022.980251)
Supplement: Supplementary file 11 [file Image_2.pdf]

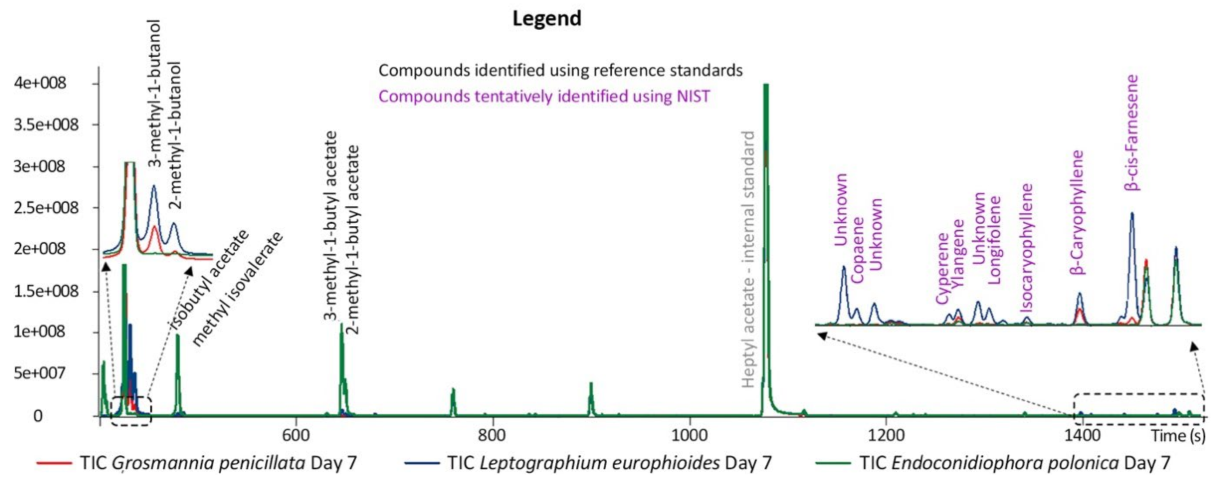

Figure S2: Total ion chromatogram (TIC) of headspace VOCs from three different fungi grown on PDA for 7 days. Green line- *E. polonica* TIC; dark blue- *L. europhioides* TIC; red- *G. penicillata* TIC
